# *N*′-(Adamantan-2-yl­idene)benzo­hydrazide

**DOI:** 10.1107/S1600536812028644

**Published:** 2012-06-30

**Authors:** Maha S. Almutairi, Ali A. El-Emam, Nasser R. El-Brollosy, Mohammed Said-Abdelbaky, Santiago García-Granda

**Affiliations:** a College of Pharmacy, King Saud University, PO Box 2457, Riyadh 11451, Saudi Arabia; b Departamento de Química Física y Analítica, Facultad de Química, Universidad de Oviedo – CINN, C/ Julián Clavería, 8, 33006 Oviedo, Asturias, Spain

## Abstract

The title mol­ecule, C_17_H_20_N_2_O, is a functionalized hydrazine with benzoyl and adamantyl substituents attached to the two hydrazine N atoms. In the crystal, mol­ecules are linked *via* N—H⋯N hydrogen bonds, forming chains propagating along the *a*-axis direction. There are also C—H⋯O, C—H⋯N and C—H⋯π inter­actions present within the chains.

## Related literature

For the biological activity of adamantane derivatives, see: Togo *et al.* (1968 ▶); Kadi *et al.* (2007 ▶, 2010 ▶); Al-Deeb *et al.* (2006 ▶); El-Emam *et al.* (2004 ▶). For the biological activity of hydrazone derivatives, see: Zheng *et al.* (2009 ▶); Moldovan *et al.* (2011 ▶). For related adamantane structures, see: Almutairi *et al.* (2012 ▶); Rouchal *et al.* (2010 ▶); El-Emam *et al.* (2012 ▶). For related cyclic ketone hydrazone structures, see: Sankar *et al.* (2010 ▶); El-Emam & Ibrahim (1991 ▶); Kia *et al.* (2009 ▶); Kadi *et al.* (2011 ▶).
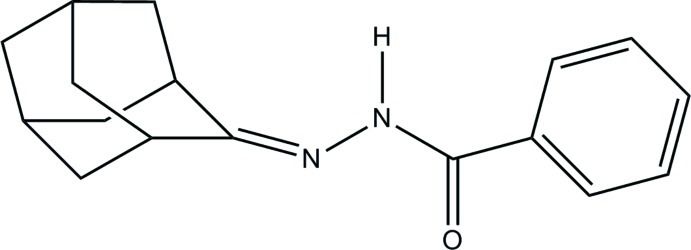


## Experimental

### 

#### Crystal data

C_17_H_20_N_2_O
*M* *_r_* = 268.35
Orthorhombic, 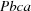

*a* = 7.9698 (3) Å
*b* = 17.5466 (8) Å
*c* = 20.1350 (8) Å
*V* = 2815.7 (2) Å^3^
*Z* = 8
Cu *K*α radiation
μ = 0.62 mm^−1^
*T* = 120 K
0.26 × 0.08 × 0.02 mm

#### Data collection

Oxford Diffraction Xcalibur Ruby Gemini diffractometer
Absorption correction: multi-scan (*CrysAlis RED*; Oxford Diffraction, 2010 ▶) *T*_min_ = 0.942, *T*_max_ = 0.988
7280 measured reflections
2634 independent reflections
1859 reflections with *I* > 2σ(*I*)
*R*_int_ = 0.052

#### Refinement

*R*[*F* ^2^ > 2σ(*F* ^2^)] = 0.051
*wR*(*F* ^2^) = 0.139
*S* = 1.03
2634 reflections
185 parameters
H atoms treated by a mixture of independent and constrained refinement
Δρ_max_ = 0.17 e Å^−3^
Δρ_min_ = −0.23 e Å^−3^

### 

Data collection: *CrysAlis CCD* (Oxford Diffraction, 2010 ▶); cell refinement: *CrysAlis CCD*; data reduction: *CrysAlis RED* (Oxford Diffraction, 2010 ▶); program(s) used to solve structure: *SIR92* (Altomare *et al.*, 1994 ▶); program(s) used to refine structure: *SHELXL97* (Sheldrick, 2008 ▶); molecular graphics: *ORTEP-3 for Windows* (Farrugia, 1997 ▶); software used to prepare material for publication: *publCIF* (Westrip, 2010 ▶).

## Supplementary Material

Crystal structure: contains datablock(s) global, I. DOI: 10.1107/S1600536812028644/su2467sup1.cif

Structure factors: contains datablock(s) I. DOI: 10.1107/S1600536812028644/su2467Isup2.hkl

Additional supplementary materials:  crystallographic information; 3D view; checkCIF report

## Figures and Tables

**Table 1:** Hydrogen-bond geometry (Å, °) *Cg*1 is the centroid of the C2–C7 ring.

*D*—H⋯*A*	*D*—H	H⋯*A*	*D*⋯*A*	*D*—H⋯*A*
N1—H1*N*⋯N2^i^	0.95 (2)	2.17 (2)	3.087 (2)	162 (2)
C3—H3⋯O1^i^	0.93	2.47	3.381 (3)	167
C9—H9⋯O1^i^	0.98	2.33	3.210 (3)	149
C9—H9⋯N2^i^	0.98	2.55	3.402 (3)	145
C15—H15*A*⋯*Cg*1^ii^	0.97	2.57	3.519 (3)	164

Symmetry codes: (i) 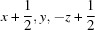; (ii) 
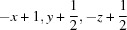
.

**Fig. 1.**
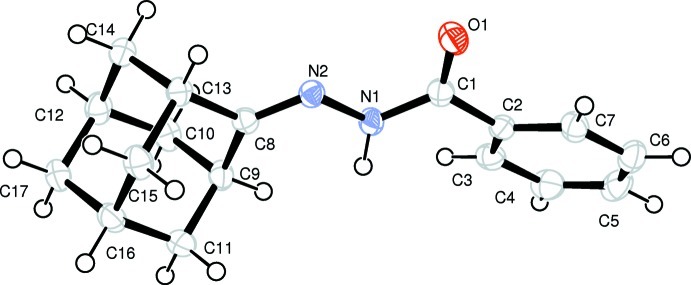
A view of the molecular structure of the title molecule with the atom numbering. Displacement ellipsoids are drawn at the 50% probability level.

## supplementary crystallographic information

### Comment

Derivatives of adamantane have long been known for their diverse biological activities including antiviral activity against the influenza (Togo *et al.*, 1968) and HIV viruses (El-Emam *et al.*, 2004). Moreover, adamantane derivatives were reported to exhibit marked antibacterial and anti-inflammatory activities (Kadi *et al.*, 2007, 2010; El-Emam & Ibrahim, 1991). In continuation to our interest in the chemical and pharmacological properties of adamantane derivatives (Almutairi *et al.*, 2012; El-Emam *et al.*, 2012), we synthesized the title compound as a precursor for potential chemotherapeutic agents. Herein, we report on the synthesis and crystal structure of the title compound.

The title molecule, Fig. 1, is a functionalized hydrazine with benzoyl and adamantyl substituents attached to the two hydrazine nitrogen atoms, N1 and N2.

In the crystal, molecules are linked via N-H···N hydrogen bonds forming chains propagating along the a axis direction. There are also C-H···O, C-H···N and C-H···π interactions present within the chains (Table 1).

### Experimental

A mixture of benzohydrazide (1.36 g, 0.01 mol), 2-adamantanone (1.5 g, 0.01 mol), in ethanol (10 ml) was heated under reflux with stirring for 4 h. On cooling, the precipitated crystalline solid was filtered, dried and recrystallized from ethanol to yield 2.52 g (94%) of the title compound as colourless needle crystals [M.p. 517-519 K]. Spectroscopic data for the title compound are given in the archived CIF.

### Refinement

The NH H-atom was located in a difference electron-density map and freely refined. The C-bound H-atoms were included in calculated positions and treated as riding atoms: C-H = 0.93, 0.97 and 0.96 Å for CH(aromatic), CH_2_ and CH(methine) H-atoms, respectively, with U_iso_(H) = 1.2U_eq_(parent C-atom).

### Crystal data

**Table:**

C_17_H_20_N_2_O	*F*(000) = 1152
*M**_r_* = 268.35	*D*_x_ = 1.266 Mg m^−^^3^
Orthorhombic, *P**b**c**a*	Cu *K*α radiation, λ = 1.54184 Å
Hall symbol: -P 2ac 2ab	Cell parameters from 1578 reflections
*a* = 7.9698 (3) Å	θ = 3.3–70.4°
*b* = 17.5466 (8) Å	µ = 0.62 mm^−^^1^
*c* = 20.1350 (8) Å	*T* = 120 K
*V* = 2815.7 (2) Å^3^	Prism, colourless
*Z* = 8	0.26 × 0.08 × 0.02 mm

### Data collection

**Table:**

Oxford Diffraction Xcalibur Ruby Gemini diffractometer	2634 independent reflections
Radiation source: Enhance (Cu) X-ray Source	1859 reflections with *I* > 2σ(*I*)
Graphite monochromator	*R*_int_ = 0.052
Detector resolution: 10.2673 pixels mm^-1^	θ_max_ = 70.4°, θ_min_ = 3.3°
ω scans	*h* = −6→9
Absorption correction: multi-scan (*CrysAlis RED*; Oxford Diffraction, 2010)	*k* = −21→20
*T*_min_ = 0.942, *T*_max_ = 0.988	*l* = −24→20
7280 measured reflections	

### Refinement

**Table:**

Refinement on *F*^2^	Primary atom site location: structure-invariant direct methods
Least-squares matrix: full	Secondary atom site location: difference Fourier map
*R*[*F*^2^ > 2σ(*F*^2^)] = 0.051	Hydrogen site location: inferred from neighbouring sites
*wR*(*F*^2^) = 0.139	H atoms treated by a mixture of independent and constrained refinement
*S* = 1.03	*w* = 1/[σ^2^(*F*_o_^2^) + (0.0662*P*)^2^] where *P* = (*F*_o_^2^ + 2*F*_c_^2^)/3
2634 reflections	(Δ/σ)_max_ < 0.001
185 parameters	Δρ_max_ = 0.17 e Å^−^^3^
0 restraints	Δρ_min_ = −0.23 e Å^−^^3^

### Special details

**Table:**

Experimental. Spectroscopic data for the title compound:^1^H NMR (CDCl_3_, 500.13MHz): δ 1.82–1.96 (m, 14H, Adamantane-H), 7.36–7.43 (m, 3H, Ar—H), 7.51–7.53 (m, 2H, Ar—H), 8.81 (s, 1H, NH). ^13^C NMR (CDCl_3_, 125.76MHz): δ 27.70, 31.82, 36.20, 37.93, 39.06, 164.47 (Adamantane-C), 127.29, 128.66, 131.73, 133.77 (Ar—C), 171.29 (C═O).
Geometry. Bond distances, angles etc. have been calculated using the rounded fractional coordinates. All su's are estimated from the variances of the (full) variance-covariance matrix. The cell esds are taken into account in the estimation of distances, angles and torsion angles
Refinement. Refinement of *F*^2^ against ALL reflections. The weighted *R*-factor *wR* and goodness of fit *S* are based on *F*^2^, conventional *R*-factors *R* are based on *F*, with *F* set to zero for negative *F*^2^. The threshold expression of *F*^2^ > σ(*F*^2^) is used only for calculating *R*-factors(gt) *etc*. and is not relevant to the choice of reflections for refinement. *R*-factors based on *F*^2^ are statistically about twice as large as those based on *F*, and *R*- factors based on ALL data will be even larger.

### Fractional atomic coordinates and isotropic or equivalent isotropic displacement parameters (Å^2^)

**Table:**

	*x*	*y*	*z*	*U*_iso_*/*U*_eq_	
O1	0.41632 (17)	0.06584 (9)	0.16083 (8)	0.0312 (5)	
N1	0.6033 (2)	0.11608 (10)	0.23403 (9)	0.0235 (5)	
N2	0.4774 (2)	0.16129 (10)	0.26108 (9)	0.0223 (5)	
C1	0.5611 (2)	0.07181 (12)	0.18124 (10)	0.0246 (6)	
C2	0.7017 (2)	0.02971 (12)	0.14858 (10)	0.0249 (6)	
C3	0.8416 (3)	0.00356 (13)	0.18307 (12)	0.0293 (6)	
C4	0.9655 (3)	−0.03725 (14)	0.14965 (14)	0.0371 (8)	
C5	0.9494 (3)	−0.05134 (15)	0.08214 (14)	0.0401 (8)	
C6	0.8098 (3)	−0.02579 (14)	0.04800 (12)	0.0369 (7)	
C7	0.6858 (3)	0.01433 (13)	0.08093 (11)	0.0293 (6)	
C8	0.5128 (2)	0.20447 (12)	0.31045 (10)	0.0220 (6)	
C9	0.6735 (3)	0.21136 (13)	0.34927 (10)	0.0264 (6)	
C10	0.6324 (3)	0.18818 (14)	0.42134 (11)	0.0310 (7)	
C11	0.7330 (3)	0.29468 (14)	0.34814 (11)	0.0313 (7)	
C12	0.4986 (3)	0.24128 (14)	0.45032 (11)	0.0296 (6)	
C13	0.3753 (2)	0.25512 (12)	0.33641 (10)	0.0237 (6)	
C14	0.3378 (3)	0.23380 (14)	0.40906 (11)	0.0291 (6)	
C15	0.4369 (3)	0.33837 (13)	0.33421 (11)	0.0296 (7)	
C16	0.5968 (3)	0.34691 (13)	0.37629 (11)	0.0288 (6)	
C17	0.5592 (3)	0.32391 (14)	0.44831 (10)	0.0279 (6)	
H1N	0.716 (3)	0.1276 (15)	0.2457 (12)	0.028 (6)*	
H3	0.85230	0.01330	0.22830	0.0350*	
H4	1.05900	−0.05500	0.17260	0.0440*	
H5	1.03280	−0.07810	0.05980	0.0480*	
H6	0.79920	−0.03560	0.00280	0.0440*	
H7	0.59160	0.03110	0.05790	0.0350*	
H9	0.75980	0.17780	0.33070	0.0320*	
H10A	0.59200	0.13600	0.42220	0.0370*	
H10B	0.73330	0.19080	0.44820	0.0370*	
H11A	0.83450	0.29970	0.37440	0.0380*	
H11B	0.75850	0.30960	0.30290	0.0380*	
H12	0.47540	0.22670	0.49640	0.0360*	
H13	0.27400	0.24930	0.30930	0.0280*	
H14A	0.25190	0.26730	0.42680	0.0350*	
H14B	0.29650	0.18190	0.41130	0.0350*	
H15A	0.46020	0.35290	0.28870	0.0360*	
H15B	0.35010	0.37190	0.35130	0.0360*	
H16	0.63530	0.39990	0.37500	0.0350*	
H17A	0.65970	0.32930	0.47510	0.0330*	
H17B	0.47350	0.35710	0.46660	0.0330*	

### Atomic displacement parameters (Å^2^)

**Table:**

	*U*^11^	*U*^22^	*U*^33^	*U*^12^	*U*^13^	*U*^23^
O1	0.0275 (7)	0.0321 (9)	0.0339 (8)	0.0024 (7)	−0.0040 (6)	−0.0082 (7)
N1	0.0219 (8)	0.0216 (9)	0.0270 (9)	0.0025 (7)	0.0013 (7)	−0.0049 (8)
N2	0.0230 (8)	0.0205 (9)	0.0235 (8)	0.0016 (7)	0.0015 (7)	−0.0002 (7)
C1	0.0276 (10)	0.0218 (11)	0.0244 (10)	0.0000 (9)	0.0005 (8)	0.0009 (9)
C2	0.0280 (10)	0.0190 (10)	0.0277 (11)	−0.0026 (8)	0.0062 (9)	−0.0018 (9)
C3	0.0274 (10)	0.0225 (11)	0.0381 (12)	−0.0009 (9)	0.0020 (9)	−0.0011 (10)
C4	0.0292 (11)	0.0298 (13)	0.0522 (15)	0.0041 (10)	0.0051 (10)	−0.0029 (12)
C5	0.0431 (13)	0.0270 (12)	0.0501 (15)	0.0032 (10)	0.0223 (12)	−0.0052 (12)
C6	0.0528 (14)	0.0248 (12)	0.0330 (12)	−0.0033 (11)	0.0161 (12)	−0.0025 (10)
C7	0.0374 (11)	0.0214 (11)	0.0290 (11)	−0.0013 (9)	0.0022 (9)	−0.0010 (9)
C8	0.0242 (9)	0.0199 (10)	0.0220 (10)	−0.0005 (8)	−0.0001 (8)	0.0015 (9)
C9	0.0245 (9)	0.0289 (12)	0.0259 (10)	0.0035 (9)	−0.0012 (9)	−0.0065 (9)
C10	0.0388 (11)	0.0284 (13)	0.0257 (11)	0.0050 (10)	−0.0087 (10)	−0.0007 (10)
C11	0.0299 (10)	0.0342 (13)	0.0298 (11)	−0.0096 (10)	0.0010 (9)	−0.0049 (10)
C12	0.0380 (11)	0.0288 (12)	0.0220 (10)	0.0016 (10)	0.0010 (9)	0.0008 (9)
C13	0.0250 (9)	0.0219 (11)	0.0242 (10)	0.0016 (8)	0.0003 (8)	−0.0014 (9)
C14	0.0307 (10)	0.0270 (12)	0.0296 (11)	−0.0017 (9)	0.0070 (9)	−0.0039 (10)
C15	0.0371 (11)	0.0235 (12)	0.0282 (11)	0.0032 (9)	−0.0021 (9)	0.0002 (9)
C16	0.0359 (11)	0.0222 (11)	0.0284 (11)	−0.0055 (10)	−0.0006 (9)	−0.0017 (9)
C17	0.0313 (10)	0.0276 (12)	0.0248 (10)	0.0014 (9)	−0.0031 (9)	−0.0060 (9)

### Geometric parameters (Å, º)

**Table:**

O1—C1	1.229 (2)	C15—C16	1.538 (3)
N1—N2	1.390 (2)	C16—C17	1.535 (3)
N1—C1	1.359 (3)	C3—H3	0.9300
N2—C8	1.281 (3)	C4—H4	0.9300
N1—H1N	0.95 (2)	C5—H5	0.9300
C1—C2	1.495 (3)	C6—H6	0.9300
C2—C7	1.394 (3)	C7—H7	0.9300
C2—C3	1.391 (3)	C9—H9	0.9800
C3—C4	1.393 (3)	C10—H10A	0.9700
C4—C5	1.388 (4)	C10—H10B	0.9700
C5—C6	1.383 (3)	C11—H11A	0.9700
C6—C7	1.383 (3)	C11—H11B	0.9700
C8—C9	1.505 (3)	C12—H12	0.9800
C8—C13	1.505 (3)	C13—H13	0.9800
C9—C11	1.537 (3)	C14—H14A	0.9700
C9—C10	1.542 (3)	C14—H14B	0.9700
C10—C12	1.532 (3)	C15—H15A	0.9700
C11—C16	1.530 (3)	C15—H15B	0.9700
C12—C17	1.529 (3)	C16—H16	0.9800
C12—C14	1.533 (3)	C17—H17A	0.9700
C13—C14	1.539 (3)	C17—H17B	0.9700
C13—C15	1.542 (3)		
			
N2—N1—C1	117.00 (15)	C5—C6—H6	120.00
N1—N2—C8	118.84 (16)	C7—C6—H6	120.00
N2—N1—H1N	117.7 (15)	C2—C7—H7	120.00
C1—N1—H1N	123.3 (15)	C6—C7—H7	120.00
N1—C1—C2	116.18 (15)	C8—C9—H9	111.00
O1—C1—C2	120.97 (18)	C10—C9—H9	110.00
O1—C1—N1	122.86 (18)	C11—C9—H9	110.00
C1—C2—C7	117.22 (17)	C9—C10—H10A	110.00
C3—C2—C7	119.77 (19)	C9—C10—H10B	110.00
C1—C2—C3	122.97 (19)	C12—C10—H10A	110.00
C2—C3—C4	119.8 (2)	C12—C10—H10B	110.00
C3—C4—C5	120.0 (2)	H10A—C10—H10B	108.00
C4—C5—C6	120.3 (2)	C9—C11—H11A	110.00
C5—C6—C7	120.1 (2)	C9—C11—H11B	110.00
C2—C7—C6	120.1 (2)	C16—C11—H11A	110.00
N2—C8—C13	117.29 (16)	C16—C11—H11B	110.00
N2—C8—C9	129.62 (18)	H11A—C11—H11B	108.00
C9—C8—C13	113.07 (17)	C10—C12—H12	109.00
C8—C9—C11	109.34 (18)	C14—C12—H12	109.00
C8—C9—C10	106.66 (18)	C17—C12—H12	109.00
C10—C9—C11	109.29 (18)	C8—C13—H13	110.00
C9—C10—C12	110.24 (19)	C14—C13—H13	110.00
C9—C11—C16	110.21 (19)	C15—C13—H13	110.00
C10—C12—C17	110.30 (19)	C12—C14—H14A	110.00
C10—C12—C14	108.88 (19)	C12—C14—H14B	110.00
C14—C12—C17	109.33 (19)	C13—C14—H14A	110.00
C8—C13—C15	108.53 (15)	C13—C14—H14B	110.00
C8—C13—C14	109.15 (17)	H14A—C14—H14B	108.00
C14—C13—C15	108.63 (17)	C13—C15—H15A	110.00
C12—C14—C13	109.39 (18)	C13—C15—H15B	110.00
C13—C15—C16	109.90 (18)	C16—C15—H15A	110.00
C11—C16—C15	109.00 (18)	C16—C15—H15B	110.00
C11—C16—C17	109.34 (19)	H15A—C15—H15B	108.00
C15—C16—C17	109.46 (19)	C11—C16—H16	110.00
C12—C17—C16	109.64 (18)	C15—C16—H16	110.00
C2—C3—H3	120.00	C17—C16—H16	110.00
C4—C3—H3	120.00	C12—C17—H17A	110.00
C3—C4—H4	120.00	C12—C17—H17B	110.00
C5—C4—H4	120.00	C16—C17—H17A	110.00
C4—C5—H5	120.00	C16—C17—H17B	110.00
C6—C5—H5	120.00	H17A—C17—H17B	108.00
			
C1—N1—N2—C8	−179.23 (19)	C9—C8—C13—C14	−59.8 (2)
N2—N1—C1—O1	−6.1 (3)	C9—C8—C13—C15	58.4 (2)
N2—N1—C1—C2	174.01 (17)	C8—C9—C10—C12	−60.3 (2)
N1—N2—C8—C9	−4.7 (3)	C11—C9—C10—C12	57.8 (2)
N1—N2—C8—C13	177.34 (17)	C8—C9—C11—C16	57.4 (2)
O1—C1—C2—C3	−148.1 (2)	C10—C9—C11—C16	−59.1 (2)
O1—C1—C2—C7	29.5 (3)	C9—C10—C12—C14	61.6 (2)
N1—C1—C2—C3	31.7 (3)	C9—C10—C12—C17	−58.3 (2)
N1—C1—C2—C7	−150.6 (2)	C9—C11—C16—C15	−59.3 (2)
C1—C2—C3—C4	178.1 (2)	C9—C11—C16—C17	60.3 (2)
C7—C2—C3—C4	0.5 (3)	C10—C12—C14—C13	−59.2 (2)
C1—C2—C7—C6	−178.7 (2)	C17—C12—C14—C13	61.3 (2)
C3—C2—C7—C6	−1.0 (3)	C10—C12—C17—C16	59.2 (2)
C2—C3—C4—C5	0.3 (4)	C14—C12—C17—C16	−60.5 (2)
C3—C4—C5—C6	−0.8 (4)	C8—C13—C14—C12	57.5 (2)
C4—C5—C6—C7	0.3 (4)	C15—C13—C14—C12	−60.7 (2)
C5—C6—C7—C2	0.5 (4)	C8—C13—C15—C16	−58.7 (2)
N2—C8—C9—C10	−117.8 (2)	C14—C13—C15—C16	59.8 (2)
N2—C8—C9—C11	124.1 (2)	C13—C15—C16—C11	60.2 (2)
C13—C8—C9—C10	60.2 (2)	C13—C15—C16—C17	−59.4 (2)
C13—C8—C9—C11	−57.9 (2)	C11—C16—C17—C12	−59.9 (2)
N2—C8—C13—C14	118.5 (2)	C15—C16—C17—C12	59.4 (2)
N2—C8—C13—C15	−123.3 (2)		

### Hydrogen-bond geometry (Å, º)

Cg1 is the centroid of the C2–C7 ring.

**Table:**

*D*—H···*A*	*D*—H	H···*A*	*D*···*A*	*D*—H···*A*
N1—H1*N*···N2^i^	0.95 (2)	2.17 (2)	3.087 (2)	162 (2)
C3—H3···O1^i^	0.93	2.47	3.381 (3)	167
C9—H9···O1^i^	0.98	2.33	3.210 (3)	149
C9—H9···N2^i^	0.98	2.55	3.402 (3)	145
C15—H15*A*···*Cg*1^ii^	0.97	2.57	3.519 (3)	164

Symmetry codes: (i) *x*+1/2, *y*, −*z*+1/2; (ii) −*x*+1, *y*+1/2, −*z*+1/2.
